# Initial Presentation of Renal Cell Carcinoma as Heart Failure Secondary to Tumor-Thrombus Extension to the Right Atrium

**DOI:** 10.7759/cureus.14537

**Published:** 2021-04-17

**Authors:** Keshav R Patel, Dilpat Kumar, Jagadeesh K Kalavakunta, FNU Warsha

**Affiliations:** 1 Medicine, Western Michigan University Homer Stryker MD School of Medicine, Kalamazoo, USA; 2 Cardiology, Ascension Borgess Medical Center, Kalamazoo, USA; 3 Internal Medicine, Interfaith Medical Center, Brooklyn, USA

**Keywords:** functional stenosis, atrial mass, renal cell carcinoma, heart failure, tumor-thrombus extension

## Abstract

Renal cell carcinoma (RCC) can invade locally through the renal vein and into the inferior vena cava (IVC) with tumor-thrombus formation reported in 5%-15% of patients. From the IVC, RCC can grow intravascularly and extend into the right atrium. We present a rare case with two uncommon findings: tumor-thrombus extension leading to a right atrial mass and initial presentation of RCC as heart failure. A 69-year-old woman presented with signs and symptoms of heart failure. Electrocardiogram was normal and the initial troponin level was mildly elevated to 0.09 ng/mL. Echocardiography revealed a dilated right atrium with a 6.9 cm x 3.8 cm echogenic mass consistent with a tumor impinging on the tricuspid valve leading to a functional stenosis. Computed tomography (CT) of the abdomen revealed a large right-sided renal mass with enlargement of the renal vein suggestive of tumor thrombus. Although the initial presentation of RCC with cardiac symptoms is surprising, this case highlights the importance of maintaining a comprehensive differential diagnosis. It also signifies the need for further imaging as not all atrial masses are cardiac tumors. Many other primary tumors - kidney, liver, lung, and thyroid - can directly invade or metastasize into the atrium by way of the vena cava.

## Introduction

Renal cell carcinoma (RCC) accounts for 90% of renal malignancies [1]. At the time of initial diagnosis of RCC, 65% of patients have localized disease, 16% of patients have regional lymph node involvement, and 16% of patients have metastatic disease [2]. RCC can invade locally through the renal vein and into the inferior vena cava (IVC) with tumor-thrombus formation reported in 5%-15% of patients [3,4]. RCC involvement of the IVC can lead to ascites, pulmonary emboli, lower extremity edema, and many other complications. From the IVC, RCC can grow intravascularly and extend into the right atrium. RCC extension to the right side of the heart is a rare complication that occurs in approximately 1% of RCC patients [3,4]. RCC is a masquerader of all malignancies and can present with a wide range of clinical signs and symptoms. The RCC classic triad of flank pain, hematuria, and a palpable abdominal mass is only found in 6-10% of patients [5]. Other common findings include scrotal varicoceles, weight loss, anemia, and erythrocytosis. Patients with RCC often develop paraneoplastic syndromes, further complicating diagnosis and management. In patients with metastatic RCC, initial presentation can vary depending on the location of the metastases. We present a rare case of RCC tumor-thrombus extension into the right atrium resulting in a 6.9 cm x 3.8 cm mass leading to severe tricuspid valve functional stenosis and initial presentation of RCC as acute heart failure.

## Case presentation

A 69-year-old Caucasian woman with a past medical history of hypertension and hypothyroidism presented with fatigue, progressive exertional dyspnea, and exertional chest pain for several weeks. The dyspnea on exertion worsened to the point where she was unable to get up from bed and walk to the bathroom without being completely out of breath (New York Heart Association [NYHA] Functional Classification III). She also endorsed orthopnea requiring three pillows to sleep at night, bilateral lower extremity swelling, and exertional chest heaviness with a left-sided pressure like sensation that lasted a few minutes and resolved with rest. On the day of presentation, she experienced non-radiating, sharp, stabbing left-sided chest pain that was different from her previous chest pain. She denied any associated cough, pleuritic/positional variant chest pain, abdominal pain, hematuria, polyuria, dysuria, or urinary incontinence.

Vitals showed a blood pressure of 119/71 mmHg, heart rate of 72 beats per minute, and respiratory rate of 20 breaths per minute. She was afebrile (97.9 F) with an oxygen saturation of 95% on room air. A pulmonary exam was significant for faint bibasilar crackles. A cardiovascular exam revealed a normal rate and regular rhythm with a 2/6 systolic ejection murmur heard at the lower left sternal border, jugular venous distension, and 2+ bilateral lower extremity edema. The chest wall was non-tender to palpation. The rest of the exam was unremarkable including an abdomen with no organomegaly.

Electrocardiogram revealed normal sinus rhythm without any ischemic abnormalities. The initial troponin level was mildly elevated at 0.09 ng/mL with a delta troponin of + 0.02 ng/mL. B-type natriuretic peptide was elevated at 1,243 pg/mL. Complete blood count (CBC) revealed a hemoglobin of 16.2 gm/dL. Complete metabolic panel (CMP) revealed an elevated creatinine of 1.5 mg/dL and mildly elevated liver tests: alkaline phosphatase of 175 IUnits/L, aspartate transaminase of 66 IUnits/L, alanine transaminase of 92 IUnits/L, and total bilirubin of 2.5 mg/dL. Prothrombin time (PT) was 15.7 seconds, activated partial thromboplastin time (APTT) was 47.8 seconds, and international normalized ratio (INR) was 1.4.

**Figure 1:**
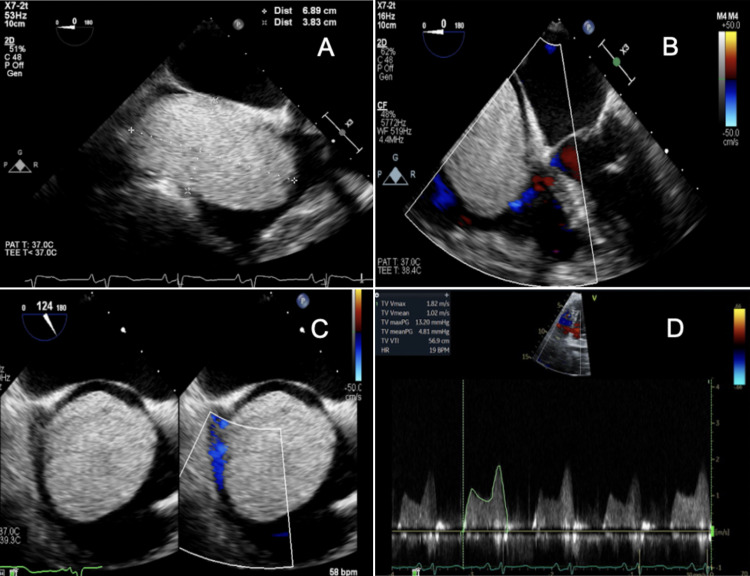
Echogenic Mass in Right Atrium Transesophageal echocardiogram (TEE) images showing the solid echogenic mass in the right atrium in different views (A-C) along with gradients across the tricuspid valve (D).

Chest x-ray revealed pulmonary vascular congestion and cardiomegaly consistent with heart failure. Transthoracic echocardiography (TTE) revealed a dilated right atrium with a 6.5 cm x 3.7 cm echogenic mass occupying the entire right atrial space and impinging on the tricuspid valve leading to tricuspid stenosis. Differentials for this mass included myxoma versus other tumors. A transesophageal echocardiogram (TEE) was obtained to better characterize the mass, which confirmed the presence of a dilated right atrium with a 6.9 cm x 3.8 cm echogenic solid mass (Figures 1A-1D). This mass was impinging on the tricuspid valve leading to severe functional stenosis (peak gradient of 13 mmHg). Both TTE and TEE showed preserved biventricular function with an estimated left ventricular ejection fraction of 60%-65%.

**Figure 2:**
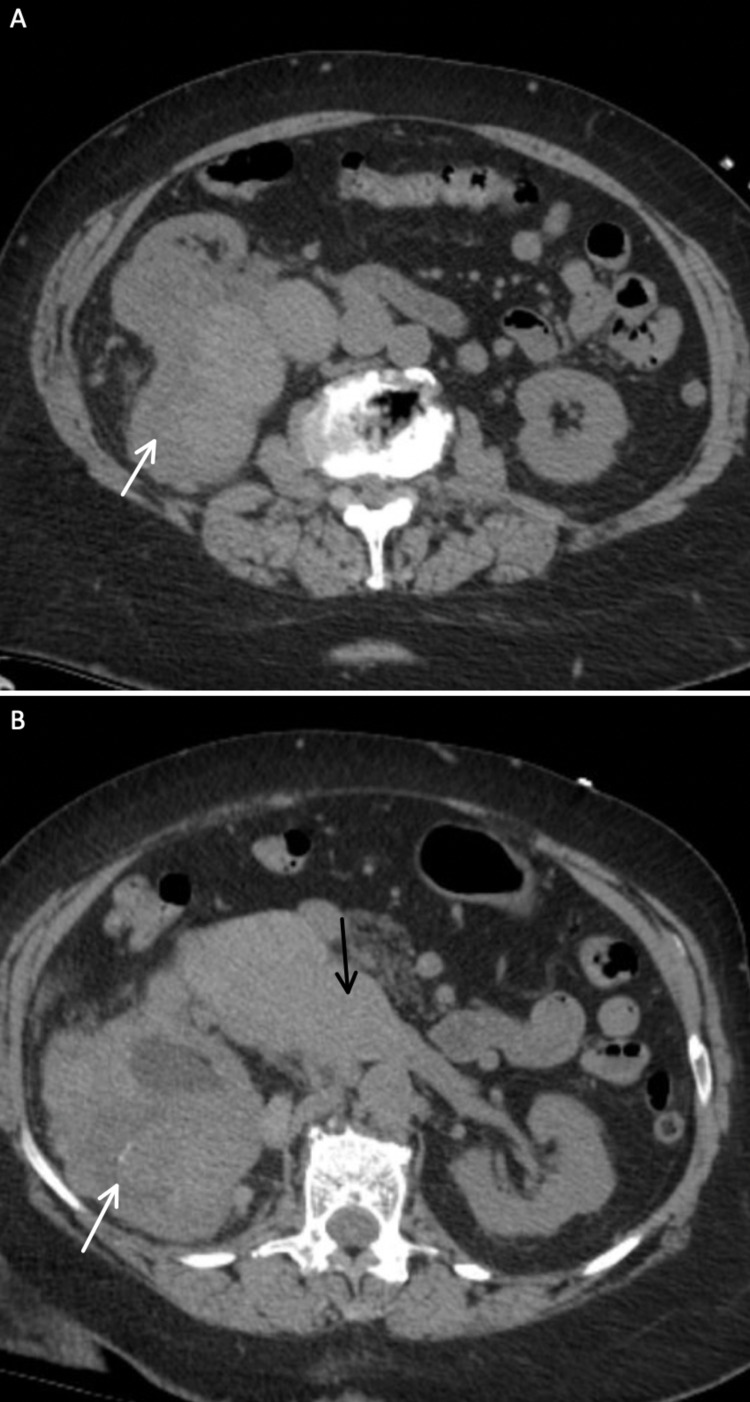
Axial View of the Right-sided Renal Mass Axial computed tomography (CT) images (A, B) showing a large multilobulated 12.8 cm x 9.3 cm solid and cystic right-sided renal mass and enlargement of the renal vein suggestive of tumor thrombus. The white arrows point towards the renal mass. The black arrow points towards the tumor thrombus extension into the renal vein and inferior vena cava.

**Figure 3:**
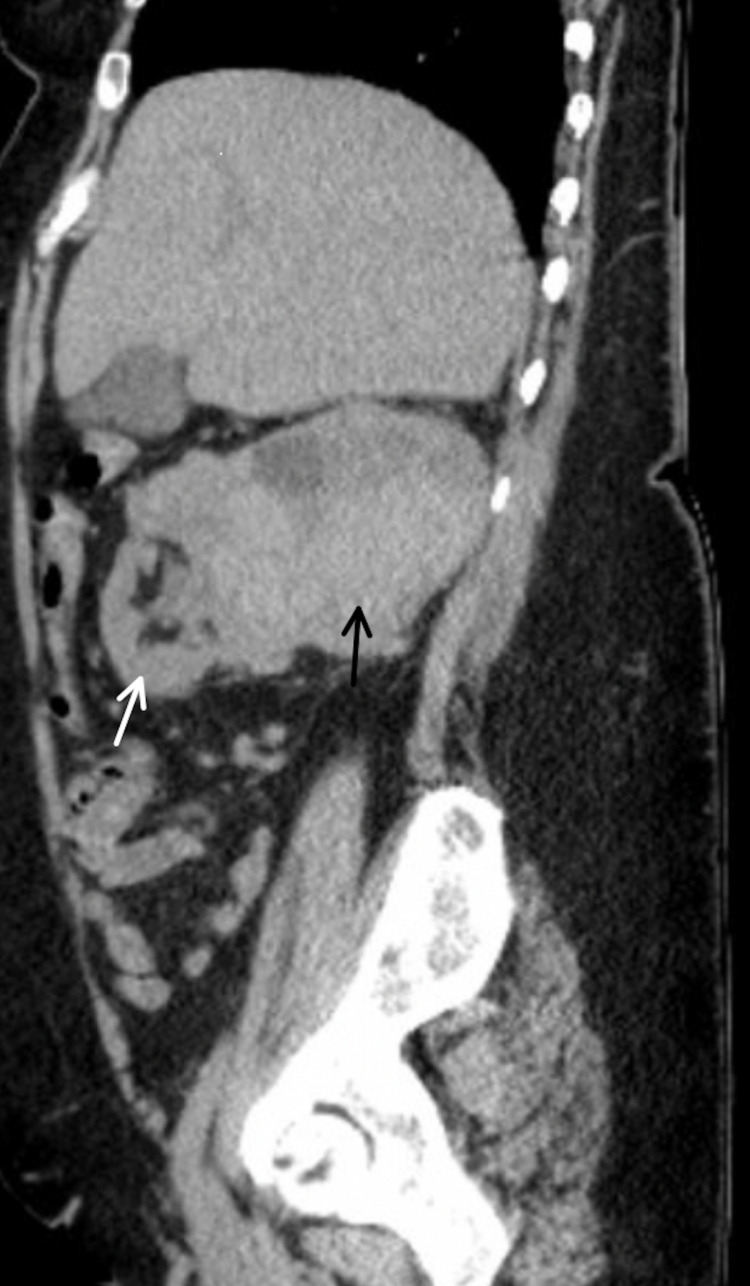
Sagittal View of the Right-sided Renal Mass Sagittal computed tomography (CT) image showing a large multilobulated 12.8 cm x 9.3 cm solid and cystic right-sided renal mass. The white arrow points towards the normal portion of the kidney. The black arrow points towards the solid and cystic renal mass.

Computed tomography (CT) of the chest/abdomen/pelvis revealed a multilobulated 12.8 cm x 9.3 cm solid and cystic right-sided renal mass that nearly replaced the right kidney with enlargement of the renal vein suggestive of tumor thrombus (Figures 2A, 2B, 3). CT also revealed enlargement of the right atrium with an abnormal density suspicious for tumor thrombus extension. Of note, CT without contrast was performed due to the elevated creatinine level. Renal ultrasound confirmed the CT findings. Urinalysis and microscopy revealed +3 red blood cells, +28 white blood cells, and +1 hyaline cast.

She initially underwent diuresis; however, she received fluids once the renal mass was found in order to maintain euvolemia. The patient’s heart failure symptoms improved, and she was referred for oncological and surgical evaluation.

## Discussion

Tumor-thrombus formation is a rare complication of RCC that occurs in approximately 5%-15% of patients [3,4]. The tumor-thrombus extended to the right atrium, resulting in a large 6.9 cm x 3.8 cm solid mass that nearly filled the right atrium and obstructed the blood flow leading to severe functional tricuspid stenosis. This mass also increased the right atrial pressure, causing septal deviation towards the left atrium. Although initial presentation of RCC with heart failure is extremely rare, RCC extension to the right side of the heart occurs in approximately 1% of RCC patients [3,4].

There are several case studies in the literature reporting functional stenosis secondary to atrial masses [6,7]. Approximately 50% of patients with left atrial myxomas have some degree of valvular obstruction and 10% have severe mitral valve functional stenosis [6,7]. However, our case is unique since the patient had moderate to severe tricuspid stenosis secondary to an atrial mass, which is commonly reported secondary to vegetations. Patients with functional stenosis can present with signs and symptoms of heart failure, as this was the case in our patient.

RCC can cause many paraneoplastic syndromes, including hypercalcemia of malignancy and erythrocytosis. Our patient had a normal calcium level; however, her hemoglobin was elevated, most likely secondary to paraneoplastic erythrocytosis or reactive erythrocytosis secondary to her dyspnea.

RCC can be diagnosed with abdominal imaging. Most patients are diagnosed with CT, but RCC can also be diagnosed with abdominal ultrasound or magnetic resonance imaging (MRI). Our patient initially underwent TTE, which revealed a mass in the right atrium, and a CT was ordered to look for possible causes of this mass. After the diagnosis of RCC is made, the patient should be evaluated for metastasis [8]. Tissue biopsy may also be beneficial to determine the subtype of RCC, which can help with prognosis and treatment [9].

The mainstay therapy for the treatment of RCC is radical tumor resection [8]. For patients with metastatic disease, tyrosine kinase inhibitors (TKIs) and threonine-serine inhibitors of mTOR (mechanistic target of rapamycin) can also be used. TKIs suppress pathways involving vascular endothelial growth factors, platelet-derived growth factors, and kinases that are involved in carcinogenesis. mTOR inhibitors prevent tumor, epithelial cell, fibroblast, and vascular smooth muscle cell proliferation. Immunotherapy that targets the programmed cell death protein 1 pathway can also be used to increase the cytotoxic response of the immune system to cancerous cells [8]. Patients with mono-metastatic or oligo-metastatic disease should be offered surgery since it is the only chance for a cure. In patients with tumor thrombus extension to the heart, radical nephrectomy with en bloc excision of the tumor thrombus extension can result in symptom resolution and improve survival [10].

The differential diagnosis for an atrial mass includes a myxoma, thrombus, vegetation, and primary or secondary neoplasm. Many other primary tumors - kidney, liver, lung, and thyroid - can directly invade the atrium or by way of the vena cava. This report emphasizes the importance of further work-up and imaging of atrial masses.

## Conclusions

Our patient presented with two uncommon findings: tumor-thrombus extension resulting in a 6.9 cm x 3.8 cm right atrial mass leading to severe tricuspid valve functional stenosis and initial presentation of RCC as acute heart failure. Although the initial presentation of RCC with cardiac symptoms is surprising, this case highlights the importance of maintaining a comprehensive differential diagnosis. It also signifies the need for further imaging as not all atrial masses are cardiac tumors.
